# Impact of Emerging Technologies on Outpatient Care Quality: Mixed Methods Study

**DOI:** 10.2196/81847

**Published:** 2026-08-19

**Authors:** Inas Al Khatib, Mahmoud Awad, Abdulrahim Shamayleh

**Affiliations:** 1 Department of Industrial Engineering, College of Engineering, American University of Sharjah, University City, Sharjah, 26666, United Arab Emirates, 971 65155555, 971 65152200

**Keywords:** digital health, telemedicine, robotic process automation, patient care quality, outpatient care, SERVQUAL

## Abstract

**Background:**

Digital transformation in health care, including telemedicine, wearable devices, robotic process automation (RPA), and electronic medical records (EMRs), is reshaping outpatient care delivery. However, consolidated evidence on how these technologies influence patient care quality (PCQ) remains limited.

**Objective:**

This study evaluated the impact of emerging digital health technologies on PCQ dimensions in outpatient clinics through a systematic literature review combined with expert perspectives.

**Methods:**

A systematic review following PRISMA (Preferred Reporting Items for Systematic Reviews and Meta-Analyses) 2020 guidelines was conducted across ABI/INFORM, ScienceDirect, MEDLINE, and Google Scholar to identify studies published between 2017 and 2022. Studies examining digital technologies and their effects on outpatient PCQ were included. After screening and eligibility assessment, 53 studies were retained for synthesis. In parallel, semistructured interviews were conducted with 19 subject matter experts from public health, health informatics, and outpatient clinical practice. Findings were thematically analyzed using SERVQUAL (Service Quality Model) dimensions: responsiveness, reliability, empathy, communication, and tangibles, and triangulated across both datasets.

**Results:**

RPA led to substantial reductions in waiting times, while telemedicine improved accessibility and continuity of care, as highlighted by 14 of 19 experts. Wearable devices enhanced remote monitoring and patient engagement but required adequate digital literacy. Several studies (n=7) reported that EMR usability challenges negatively affected patient-physician communication. Overall, improvements were most evident in responsiveness and communication, while empathy and patient-centeredness remained comparatively weaker without targeted training and workflow adjustments.

**Conclusions:**

Emerging digital health technologies can enhance outpatient PCQ, particularly in operational efficiency, access to care, and communication. This review is innovative in that it integrates evidence across telemedicine, wearable monitoring, RPA, and EMRs within a unified SERVQUAL framework, offering practical guidance for implementing patient-centered digital health strategies in outpatient settings.

## Introduction

### Background

The surge in health care demand directly correlates with expenses and resource allocation, underscoring the imperative to prioritize enhancing service quality on the management agendas of health care operators. This prioritization is crucial due to its profound impact on patients’ perceived value, satisfaction levels, and loyalty [1]. A comprehensive comprehension of service quality in health care commences with grasping its conceptualization. Despite its importance, there is no unified common understanding of patient care quality (PCQ). Instead, there are several interpretations, starting with a complex and abstract definition that defies standardization and quantification, acknowledged as fundamental to customer satisfaction and contentment [2]. Second, it involves clients’ perceptions and assessments of the service, elucidating the disparity between expectations and experiences [3]. This perspective resonates with those defining service quality as the differential between clients’ initial expectations and those developed postservice interaction [4]. Third, service quality encompasses a service provider’s efficacy in satisfying consumers effectively, thereby fostering heightened profits, market share, sustainable customer satisfaction, and corporate marketing prowess [5,6]. Moreover, service quality is intricately intertwined with service delivery, evaluated through diverse determinants governing performance and consumer benefits [7]. Finally, customers significantly influence service delivery, assessing quality via a 5-gap model encompassing knowledge, standards, communication, delivery, and the differential between expected and actual service quality [2].

There is an increasing demand for outpatient services, leading to an increase in the patient volumes seen, increased waiting times through the patient journeys, overworked caregivers, and increased overtime hours worked. Evaluating the patient’s satisfaction with the quality of outpatient clinic service delivery [8]. Health care operators realized the need to change the way they work [9]. Digital transformation improves 4 key operational decision-making areas: patient flow, staffing, scheduling, and supply chain [10].

**Figure 1.**
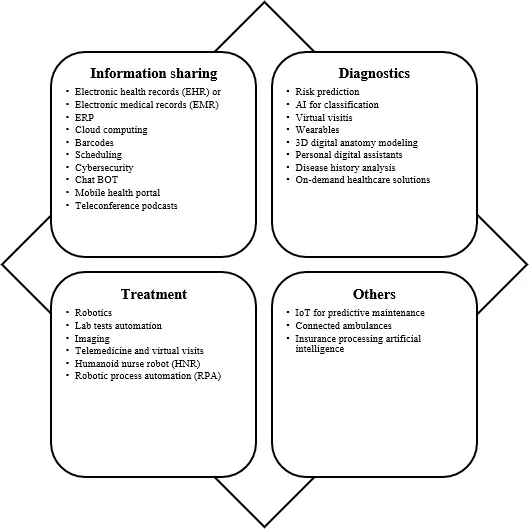
Available technologies for health care. ERP: enterprise resource planning; IoT: Internet of Things.

To address health care challenges and intense competition, technology adaptation has become a priority for decision-makers. Figure 1 illustrates some of the technologies used by the health care sector to tackle these various challenges. Digital transformation involves a process aimed at enhancing an entity by instigating significant changes to its attributes through the integration of information, computing, communication, and connectivity technologies [11]. As highlighted by Allen [12], it serves as a pivotal step in preparing for a consumer-centric “Future of Health.” Cloud adoption, including Platform as a Service (PaaS), Infrastructure as a Service (IaaS), and Software as a Service (SaaS) [12], along with advancements in AI and the Internet of Things (IoT), are actively driving global digital transformation efforts [13]. Esteemed health systems perceive digital transformation to become more customer-oriented while simultaneously evolving their operations, work culture, and technological landscape. This includes outpatient clinics leveraging digital capabilities to fundamentally reshape their client relationships, focusing on incremental milestones to deliver value. However, successful transformation faces several challenges, including talent acquisition, data management, establishing key performance indicators, and budget allocation. Notably, having executive leadership dedicated to championing digital transformation is essential for success [14]. Barriers in health care digital transformation encompass the cybersecurity workforce shortage and reliance on legacy systems [13]. Key technologies driving digital transformation include automation, connected ambulances, on-demand health care solutions, telemedicine, virtual visits, patient portals, health care wearables, and disease history analysis. These innovations aim to streamline workflows for clinicians and hospitals, ensuring accurate patient data and health performance indicators to facilitate more effective treatment plans in shorter timeframes. Hospitals stand to benefit from optimized workflows, improved client interaction, cost reduction, secure electronic medical records (EMR) databases, and enhanced communication with medical staff [15]. Patients, on the other hand, gain access to personalized health care services, simplified access to personal health records, convenient appointment scheduling, improved online communication with doctors, and real-time tracking of crucial health metrics [16].

### Rationale

Consequently, the primary focus of this review article revolves around investigating the impact of adopting emerging technologies on PCQ, encompassing both clinical and nonclinical dimensions, for assessment and monitoring purposes from the perspective of health practitioners operating within an outpatient setting to determine their impact on patient experience. These dimensions, as highlighted by Abbasi-Moghaddam et al [17] and Berwick [18], include safety, effectiveness, patient-centeredness, timeliness, efficiency, and equity. Assessing health care quality proves challenging due to its intangible nature, with evaluations varying based on factors such as the specialization, size, and complexity of health care organizations [5]. It involves strategic decision-making across various disciplines within health care, with perceptions differing among different professional fields [19].

Substandard health care quality can lead to patient dissatisfaction, stress, and despair [7]. It is estimated that poor-quality health care contributes significantly to health care costs, accounting for approximately 30%, owing to reduced productivity, wasteful clinical practices, high rates of medical errors, and costly health issues stemming from individual behaviors [20]. The World Health Organization [21] identifies factors such as inaccurate diagnosis, unnecessary treatments, unsafe clinical practices, and inadequately trained health care professionals as drivers of escalating illness burden and health care costs, commonly referred to as the cost of poor quality (COPQ). The broader economic and social costs of substandard care amount to trillions of dollars annually on a global scale due to long-term disability and lost productivity.

Despite extensive research exploring the determinants of health care quality and their impact on various operational aspects, there remains a gap in understanding the evolution of these determinants over time and the role of emerging technology adoption in enhancing PCQ. This research seeks to address this gap by examining whether investments in emerging technologies aimed at improving outpatient clinic quality determinants result in positive patient experience from the perspective of health care professionals operating in outpatient clinic settings. By examining a wide range of published articles and existing literature, this review aims to provide a comprehensive understanding of patients’ perspectives. Specifically, the integration of technologies such as robotic process automation (RPA), telemedicine and virtual visits, mobile health (mHealth) portal, videoconferencing, other educational technology, 3D digital anatomy modeling, insurance processing AI, health care wearables, personal digital assistants (PDAs), disease history analysis, teleconference podcasts, EMRs, on-demand health care solutions, and connected ambulance is being evaluated. These technologies have the potential to streamline processes, improve efficiency, and enhance the overall patient experience throughout their treatment journey.

Thus, the main research question to be explored through further methodologies is does the use of emerging technologies in outpatient clinics lead to better quality determinants and enhanced patient experiences?

This study was guided by the following hypothesis: emerging technology adoption and integration enhances all dimensions of PCQ in outpatient health clinics.

Recent review studies have examined the role of individual digital health technologies, such as telemedicine, wearable monitoring devices, and EMRs, in improving health care delivery and patient outcomes. However, these reviews typically focus on single technologies or specific clinical contexts, providing limited insight into how multiple digital health innovations collectively influence PCQ in outpatient settings. As digital transformation increasingly involves the simultaneous implementation of interconnected technologies, a more integrated understanding of their combined effects on health care service quality is needed. To address this gap, this systematic review synthesizes evidence across multiple digital health domains and evaluates their impact on PCQ using SERVQUAL (Service Quality Model) dimensions. By consolidating fragmented evidence and incorporating expert perspectives, this review offers a more comprehensive assessment of how emerging digital technologies shape our PCQ and provides practical insights for health care decision-makers [22-25].

### Objectives

To answer the research question and test the research hypothesis, the following objectives are identified:

1. Identify the dimensions that influence the perception of PCQ in outpatient clinics
2. Assess the present emerging technology endeavors integrated into determinants of health service quality in outpatient clinics
3. Authenticate the favorable patient experience stemming from emerging technology initiatives implemented within determinants of health service quality in outpatient clinics

This research will directly benefit health care providers and quality experts, particularly those working in hospitals with outpatient clinics, medical centers, or satellite clinics. Additionally, academic scholars, research scholars, accreditation professionals, continuous improvement specialists, regulatory bodies, and occupational health and safety professionals will also find value in this study.

## Methods

### Overview

This study followed the PRISMA (Preferred Reporting Items for Systematic Reviews and Meta-Analyses) 2020 guidelines for systematic reviews (Checklist 1) and the COREQ (Consolidated Criteria for Reporting Qualitative Research) checklist for expert interviews (Checklist 2). The methodology followed in this research consists of 2 main steps: systematic literature review (SLR) and subject matter experts (SMEs) interviews. The 2 steps were intentionally designed to provide complementary perspectives on the central research question, each contributing a distinct layer of insight. The systematic literature review established the theoretical foundation, identifying key quality dimensions across health care technology interventions. The expert interviews offered contextual validation from stakeholders actively involved in implementation. The SLR is used to gather secondary data and analyze its relevance to the study objectives. This method was selected due to its ability to offer a comprehensive, evidence-based synthesis of prior research, aligning with best practices recommended by Nunn and Chang [26] and Siddaway et al [27]. Systematic reviews are particularly well-suited for health care–related inquiries because they apply a transparent, replicable methodology that ensures rigor in identifying, integrating, and evaluating diverse sources.

### Eligibility Criteria

Studies were selected according to predefined inclusion and exclusion criteria to ensure relevance, methodological consistency, and alignment with the review objectives, as shown in Textbox 1.

Textbox 1. **Inclusion and exclusion criteria.**

**Inclusion criteria**

- Outpatient or ambulatory care setting
- Use of emerging technologies (telemedicine, robotic process automation, electronic medical record or electronic health record, AI, wearables, and educational tech)
- Reported patient care quality or satisfaction outcomes
- Peer-reviewed empirical or systematic review studies

**Exclusion criteria**

- Inpatient or hospital-only focus
- Non–peer-reviewed (editorials and conference abstracts)
- Technology not linked to patient care or service quality

### Information Sources

A systematic search was conducted across 7 major databases: ABI/INFORM Global, ScienceDirect, MEDLINE (via PubMed), Google Scholar, Embase, Cochrane Library, and CINAHL*.* Studies published between 2017 and 2022 were prioritized. The search strategy was designed to capture digital health innovations in outpatient care and their impact on patient experience, quality, and satisfaction. Database-specific search syntax, MeSH and Emtree terms, synonyms, and field codes were used. To ensure validity and reliability, studies published between 2017 and 2022 were prioritized.

### Search Strategy

#### Overview

The search included three key concept clusters:

1. Digital health technologies: such as telemedicine, EMR and electronic health record (EHR), AI, mHealth apps, and wearables
2. Outpatient care: such as ambulatory care, outpatient clinic, and primary care
3. Patient-centered outcomes: patient experience, patient satisfaction, and quality of care

Boolean operators AND and OR combined these clusters, and truncation and wildcards were applied where supported. For each database, search strings were adapted to reflect its indexing and search syntax. Detailed search strings and database queries are provided in this section.

#### Boolean Search Strings Across Databases

The following Boolean search strings were used for the database searches:

1. (“digital health” OR “eHealth” OR “telehealth” OR “mobile health” OR “mHealth” OR “health IT” OR “digital innovation”) AND (“outpatient care” OR “ambulatory care” OR “primary care clinic” OR “ambulatory services”) AND (“patient experience” OR “patient satisfaction” OR “quality of care” OR “patient-centered care”)
2. (“telemedicine” OR “virtual care” OR “remote consultation” OR “video consultation”) AND (“patient satisfaction” OR “patient perception” OR “patient experience”)
3. (“robotic process automation” OR “RPA”) AND (“healthcare” OR “medical services”) AND (“outpatient” OR “ambulatory care”)
4. (“electronic health record” OR “EHR” OR “EMR” OR “HER”) AND (“patient-centered care” OR “quality of care” OR “service quality”)
5. (“wearable devices” OR “healthcare wearables” OR “mHealth apps” OR “mobile health portal” OR “smartwatch health app”) AND (“outpatient clinic” OR “ambulatory services”)
6. (“artificial intelligence” OR “AI” OR “machine learning” OR “deep learning”) AND (“insurance processing” OR “clinical decision support” OR “diagnostic support”) AND (“outpatient” OR “ambulatory care”)
7. (“3D anatomy modeling” OR “educational technology” OR “digital simulation” OR “virtual reality training”) AND (“patient education” OR “health education”) AND (“outpatient setting” OR “ambulatory clinic”)
8. (“personal digital assistant” OR “clinical decision support system” OR “mobile clinical assistant”) AND (“physician competence” OR “clinical performance”) AND (“outpatient” OR “ambulatory care”)

#### Database-Specific Notes

The following provides database-specific notes on searches.

- ABI/INFORM Global: all Boolean strings applied with limits, including English language, peer-reviewed journals, and publication years 2017‐2022.
- ScienceDirect: used Boolean strings with filters for research articles and review articles, publication years 2017‐2022.
- Google Scholar: keywords applied sequentially due to limitations on advanced Boolean combinations; the first 200 results for each query were screened.
- Medline (PubMed):
  - Used MeSH terms: “Telemedicine”[MeSH], “Electronic Health Records”[MeSH], “Patient Satisfaction”[MeSH]
  - Combined with title and abstract searches: (digital health[tiab] OR eHealth[tiab] OR mHealth[tiab])
  - Filters: English language, human studies, and publication years 2017‐2022
- Embase:
  - Used Emtree terms: “telemedicine”/exp, “electronic health record”/exp, “patient satisfaction”/exp
  - Field modifiers: ti,ab for title/abstract
  - Filters: English language and publication years 2017‐2022
- ABI/INFORM Global, ScienceDirect, CINAHL, Cochrane Library, and Google Scholar:
  - Boolean operators applied with syntax adjustments per database
  - First 200 results screened in Google Scholar due to limits
- Notes on reproducibility:
  - All database-specific queries, including MeSH and Emtree terms, truncation, and field codes, are fully documented
  - Any item not applicable to a database was noted (such as MeSH terms in Google Scholar).

This detailed reporting ensures transparency and reproducibility of our search methodology, in accordance with PRISMA-S (Preferred Reporting Items for Systematic Reviews and Meta-Analyses literature search extension) guidelines.

### Data Synthesis

#### Overview

To supplement the literature review, semistructured interviews were conducted with 19 SMEs across the fields of public health, health care informatics, and blockchain-based health technology. The experts were selected using purposive sampling, ensuring diversity in terms of geographic location (United Arab Emirates, Europe, and North America), professional role (policymakers, IT developers, and medical practitioners), and years of experience (ranging from 8 to 25 years). The interview protocol covered themes such as data privacy, trust in decentralized systems, donor engagement, and integration challenges within health care infrastructures. Responses were coded thematically and triangulated with findings from the literature review.

This study was reviewed and approved by the Institutional Review Committee (IRB) at the American University of Sharjah in the United Arab Emirates. All expert participants provided written informed consent for interview participation.

#### Risk of Bias Assessment

To ensure methodological rigor and transparency, the risk of bias of all included studies was assessed using established appraisal tools appropriate to the study design. Randomized studies were evaluated using the Cochrane Risk of Bias Tool 2*,* while observational studies were assessed using the Newcastle-Ottawa Scale. For nonrandomized intervention studies, the ROBINS-I was applied where relevant. Two reviewers independently conducted the quality and risk-of-bias assessments, and any disagreements were resolved through discussion and consensus to ensure consistency and reliability. The results of the risk-of-bias evaluation were documented in summary tables and considered during the synthesis and interpretation of findings. Studies identified as having a higher risk of bias were carefully examined during analysis to ensure that conclusions regarding the impact of digital health technologies on outpatient PCQ were not disproportionately influenced by lower-quality evidence.

#### Screening and Selection

Screening followed PRISMA guidelines, with a flow diagram illustrating identification, screening, eligibility, and inclusion steps.

#### Data Collection

To organize the findings of the literature and interview questions, several models focused on suggesting dimensions to measure PCQ were reviewed. The SERVQUAL model was selected for this study due to its widespread application, simplicity, and robust structure in assessing service quality across diverse sectors, including health care [28]. It operationalizes service quality through 5 distinct dimensions: tangibility, reliability, responsiveness, assurance, and empathy, which align closely with patient-centered care indicators [29]. Several researchers advocated for implementing the SERVQUAL model as a robust method for assessing service quality in outpatient health care facilities [2]. While alternative models such as the HEALTHQUAL [30] and HCAHPS [31] also assess health care quality, SERVQUAL offers a more generalized and flexible structure that allows for comparative analysis across settings and time, making it suitable for exploratory research in dynamic or cross-cultural health care environments [32]. Furthermore, unlike HCAHPS, which is limited to inpatient experiences and largely administrative reporting, SERVQUAL captures perceived service gaps directly from users, making it more relevant for studies focused on user satisfaction and perceived service deficiencies [33]. Thus, its adaptability, empirical grounding, and comparative advantage in measuring perceived service quality support its use in this context.

The SERVQUAL model is a widely used framework for measuring and managing service quality across various industries. It was developed based on the premise that customers’ perceptions of service quality are influenced by the gap between their expectations and their actual experiences with a service. SERVQUAL breaks down service quality into 5 dimensions, often represented by the acronym RATER. First, reliability, which refers to the ability of a service provider to deliver its services consistently and accurately. Customers expect services to be performed dependably and accurately. Second, assurance relates to the knowledge and courtesy of employees and their ability to inspire trust and confidence. It includes factors such as employees’ competence, credibility, courtesy, and ability to convey trust and confidence. Third, tangibles refer to the physical evidence and appearance of facilities, equipment, personnel, and communication materials. This dimension encompasses the appearance of physical facilities, equipment, personnel, and communication materials. Fourth, empathy which involves the degree of caring, individualized attention, and understanding that the service provider gives to its customers. It relates to the provider’s ability to understand and empathize with customers’ needs and concerns. Fifth, responsiveness, which refers to the willingness of the service provider to help customers and provide prompt service. It involves aspects such as a willingness to assist customers promptly, attentiveness, and responsiveness to customer requests or inquiries.

The SERVQUAL model is typically assessed using a survey questionnaire that measures customers’ perceptions and expectations across these 5 dimensions. Customers are asked to rate both their expectations of an ideal service (ie, how they believe a service should be) and their perceptions of the actual service received. The difference between customers’ expectations and perceptions provides insights into the gaps in service quality. This survey questionnaire was used in interviewing 23 SMEs, yet only feedback from 19 experts operating in an outpatient clinic setting was considered. Those respondents operate in different capacities as outlined in Table 1 below.

Through this research review paper, ethical measures are followed in terms of honesty in data reporting, objectivity, confidentiality, integrity and sincerity, human error and negligence avoidance, and transparency in results sharing.

**Table 1.** Interviewed SME^a^ demographics.

Job title	Age bracket (years)	Years of experience
Continuous improvement project manager	40‐49	11‐15
Receptionist	30‐39	6‐10
Clinic information officer	30‐39	3‐5
Customer care associate	30‐39	3‐5
Administrator	40‐49	6‐10
Receptionist	30‐39	11‐15
Charge nurse (Aviation Medicine Clinic)	40‐49	16‐20
Officer	40‐49	≥20
Registered nurse	50‐59	≥20
Doctor	40‐49	16‐20
Manager clinical systems	40‐49	11‐15
Registered nurse	30‐39	6‐10
Registered nurse	30‐39	11‐15
In-flight nurse	30‐39	6‐10
Aviation nurse	40‐49	11‐15
Executive director, nursing services	40‐49	≥20
Respiratory therapist	50‐59	≥20
Practical nurse	30‐39	6‐10
Registered nurse	40‐49	≥20

a SME: subject matter expert.

### Ethical Considerations

This study was approved by the IRB Committee of the American University of Sharjah in the United Arab Emirates (Protocol Number 25-091). Written informed consent was obtained from all interview participants.

## Results

**Figure 2.**
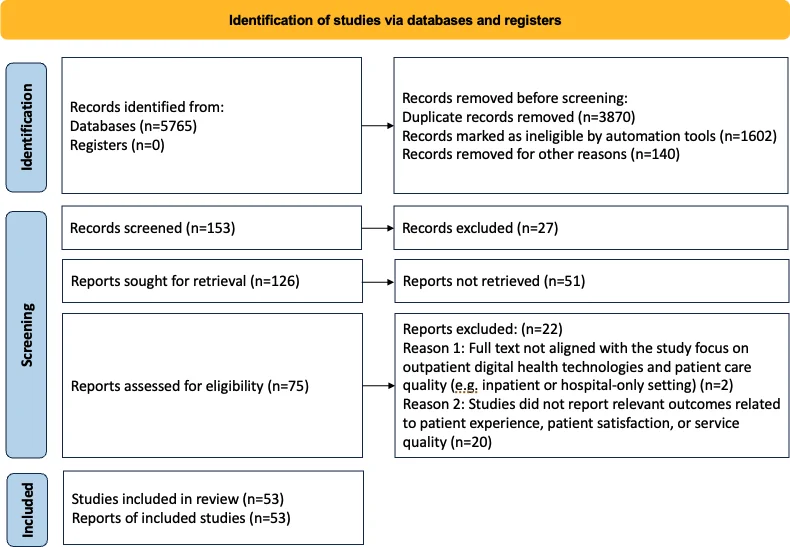
PRISMA (Preferred Reporting Items for Systematic Reviews and Meta-Analyses) 2020 flow diagram.

The initial search generated 5765 records, which were screened by title and abstract. After applying inclusion and exclusion criteria and assessing full texts, 53 relevant articles published between 2017 and 2022 were included (Figure 2).

Literature reviewed part of the SLR consists of research studies and case studies. The review of the case studies was not to offer an exhaustive ethnographic account, but rather to illustrate the practical implications of identified trends. An augmented version of SERVQUAL was applied selectively as a structuring tool during the literature synthesis phase to help organize findings under recognized quality dimensions. Other dimensions such as trust, data governance, and interoperability emerged more prominently in expert and case study findings, which necessitated a broader, more adaptive analytical approach. This divergence reflects the evolving nature of health care service delivery in the digital era and underscores the need for hybrid evaluative frameworks that go beyond traditional service quality models.

Interview respondents confirmed that safety is the most important quality dimension, followed by patient-centeredness from a patient perspective, as demonstrated in Table 2. The study assumes that emerging technologies generally yield positive outcomes across quality dimensions for a favorable patient experience. Tables 3 and 4 provide a summary of the SMEs’ feedback on the impact of technology on organizational improvement objectives and PCQ dimensions, respectively. Some of the organizational objectives are focused on PCQ, such as overall satisfaction, patient education, and patient information sharing.

**Table 2.** Which quality dimension is considered the most important from a patient perspective?

Dimension	Responses, n
Not disclosed	1
Effectiveness	2
Efficiency	1
Equity	3
Patient-centeredness	4
Safety	5
Timeliness	3
Total	19

**Table 3.** Technology impact on organizational improvement objectives.

Technology	Organizational improvement category						
	Patient satisfaction	Caregiver perception	Data collection	Productivity	Reduce cost	Patient education	Patient information sharing
Robotic process automation	1-PI^a^	1-PI	1-PI	2-SPI^b^	2-SPI	1-PI	1-PI
Telemedicine and virtual visits	1-PI	1-PI	0-NI^c^	1-PI	2-SPI^d^	1-PI	1-PI
Robotics	1-PI	1-PI	1-PI	1-PI	1-PI	1-PI	1-PI
Mobile health portal	2-SPI	1-PI	1-PI	2-SPI	1-PI	1-PI	1-PI
Videoconferencing	1-PI	1-PI	1-PI	1-PI	1-PI	1-PI	1-PI
Other educational technology	2-SPI	2-SPI^d^	1-PI	1-PI	1-PI	2-SPI^d^	1-PI
3D digital anatomy modeling	2-SPI^d^	2-SPI^d^	2-SPI^d^	0-NI	2-SPI	2-SPI^d^	2-SPI
Insurance processing AI	1-PI	1-PI	1-PI	1-PI	1-PI	1-PI	1-PI
Health care wearables	1-PI	1-PI	1-PI	1-PI	1-PI	1-PI	1-PI
Personal digital assistants	1-PI	1-PI	1-PI	1-PI	1-PI	1-PI	1-PI
Disease history analysis	2-SPI	2-SPI^d^	2-SPI	1-PI	1-PI	2-SPI	2-SPI
Teleconference podcasts	1-PI	1-PI	1-PI	1-PI	1-PI	1-PI	1-PI
Electronic medical records	2-SPI	2-SPI	2-SPI	2-SPI	2-SPI	2-SPI^d^	2-SPI
On-demand health care solutions	2-SPI^d^	2-SPI^d^	2-SPI^d^	2-SPI^d^	1-PI	2-SPI^d^	1-PI
Connected ambulance	2-SPI	2-SPI	2-SPI	2-SPI^d^	1-PI	1-PI^d^	2-SPI

a PI: positive impact.

b SPI: strong positive impact.

c NI: no impact.

d When there is a tie in feedback, the highest scale is considered (eg, equal representation of 2-SPI, 1-SPI, and O-NI).

**Table 4.** Technology impact on PCQ^a^ dimensions legend.

Technology	Quality dimension								
	Professional competence and credibility	Service reliability and assurance	Patient-centeredness and empathy	Responsiveness, waiting, and service time	Nurse care	Access to care/care equality	Service environment and cleanliness	Communication	Technical support
Robotic process automation	2-SPI^b^	2-SPI	0-NI^c^	2-SPI	1-PI^d^	2-SPI	1-PI	1-PI	2-SPI
Telemedicine and virtual visits	1-PI	1-PI	1-PI	2-SPI^e^	1-PI	1-PI	1-PI	1-PI	1-PI
Robotics	2-SPI	1-PI	2-SPI^e^	1-PI	1-PI	2-SPI^e^	1-PI	1-PI	2-SPI^e^
Mobile health portal	2-SPI^e^	1-PI	2-SPI^e^	1-PI	1-PI	1-PI	1-PI	2-SPI	2-SPI
Videoconferencing	1-PI	1-PI	1-PI	1-PI	1-PI	1-PI	1-PI	2-SPI^e^	1-PI
Other educational technology	1-PI	1-PI	1-PI	1-PI	2-SPI	2-SPI	1-PI	2-SPI^e^	2-SPI
3D digital anatomy modeling	0-NI	1-PI^e^	1-PI^e^	0-NI	1-PI	0-NI	1-PI^e^	1-PI	1-PI
Insurance processing AI	2-SPI^e^	2-SPI	2-SPI^e^	2-SPI	2-SPI^e^	2-SPI	2-SPI*^e^	2-SPI^e^	2-SPI^e^
Health care wearables	1-PI	1-PI	0-NI	0-NI	1-PI	1-PI	1-PI	1-PI	1-PI
Personal digital assistants	1-PI	1-PI	0-NI	1-PI	1-PI	1-PI	1-PI	1-PI	1-PI
Disease history analysis	2-SPI	2-SPI	2-SPI	2-SPI	2-SPI	2-SPI^e^	0-NI	2-SPI	2-SPI
Teleconference podcasts	1-PI	1-PI^e^	1-PI^e^	0-NI	1-PI	1-PI^e^	1-PI	1-PI	1-PI
Electronic medical records	2-SPI	2-SPI	2-SPI	2-SPI	2-SPI	2-SPI	2-SPI	2-SPI	2-SPI
On-demand solutions	2-SPI	2-SPI^e^	2-SPI	2-SPI	2-SPI	2-SPI	1-PI	2-SPI	2-SPI
Connected ambulance	0-NI	2-SPI	2-SPI	2-SPI	1-PI	2-SPI	2-SPI^e^	1-PI	2-SPI^e^

a PCQ: patient care quality.

b SPI: strong positive impact.

c NI: no impact.

d PI: positive impact.

e When there is a tie in feedback, the highest scale is considered (eg, equal representation of 2-SPI, 1-SPI, and O-NI).

One of the major findings both the SLR and interviews revealed is the patient-centeredness. Patients differ significantly in their attitudes and preferences; some may be submissive, readily accepting prescribed treatments, while others might be technology enthusiasts who prefer to engage with digital health tools. Additionally, there are those who might challenge medical advice and seek more information or alternative options. Recognizing and addressing these variations is crucial for delivering personalized and effective care, especially if technology is used [34].

## Discussion

### Principal Findings

**Figure 3.**
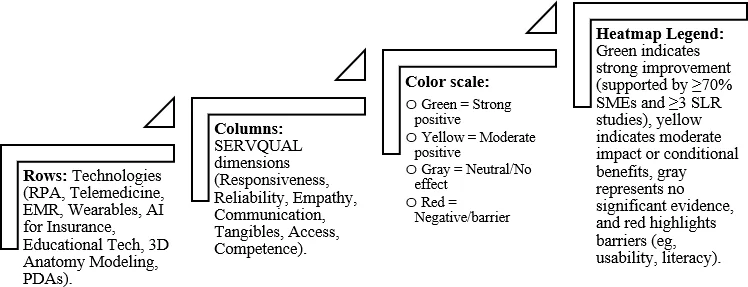
Heatmap of technology impact on SERVQUAL dimensions. EMR: electronic medical record; PDA: personal digital assistant; RPA: robotic process automation; SERVQUAL: Service Quality Model; SLR: systematic literature review; SME: subject matter expert.

In the next sections, the results are discussed in two stages: (1) SLR findings structured by SERVQUAL dimensions, and (2) SME insights triangulated with literature trends. Key quantitative outcomes from reviewed studies (eg, reductions in waiting time and improved satisfaction scores) are tabulated, while qualitative themes emphasize usability barriers, empathy gaps in telemedicine, and digital literacy challenges. Findings from the SLR and expert interviews (n=19) were mapped against SERVQUAL dimensions to illustrate the impact of emerging technologies. Figure 3 presents a heatmap showing technology influence across dimensions, while Table 1 details key quantitative effects and expert agreement rates.

This study examined whether the adoption of emerging digital health technologies improves PCQ in outpatient clinics. By synthesizing findings from 53 peer-reviewed studies and triangulating them with insights from 19 SMEs, the results indicate that digital technologies generally enhance several dimensions of outpatient service quality. The most consistent improvements were observed in responsiveness, communication, and operational efficiency through technologies such as robotic process automation, telemedicine, and EMRs. However, the findings also highlight that technological implementation alone does not automatically improve patient-centeredness or empathy, and that factors such as usability, digital literacy, and clinician training influence the extent to which technology translates into improved patient experiences. Overall, the results partially support the study hypothesis that emerging technologies enhance PCQ dimensions, while also emphasizing that human factors and organizational readiness remain critical to achieving patient-centered outcomes.

### Dimension 1: Responsiveness and Timeliness (Waiting Time and Service Time)

Excessive patient waiting time at outpatient clinics yields undesired levels of patient satisfaction and demands the need to be efficiently managed from a cost and duration perspective [8]. Patients perceive being at the receiving end of service quality when there is an patients strictly follow their scheduled appointments and doctors are available according to the promised time communicated to them [35].

The most promising technologies cited or appraised by SMEs are RPA, EHR, and connected ambulances. RPA relies on a combination of workflow, business rules, and presentation layer integration with information systems to act as a semi-intelligent user of the system, which can be used in preauthorization, updating patient records, and billing (repetitive tasks) [36]. RPA can be used for temporary or permanent outpatient clinics [37]. documented the use of RPA and its positive impact on the waiting time for an urgent care service for COVID-19. RPA was used to develop a web platform digital solution that uses modern Hode-JS, RESTful, and SOAP technologies to process data entries by the nurses, in addition to automating the administrative process at the EHR, replacing human intervention when interpreting data entry. The queuing system number generated by the EHR for the patient and displayed on a visual display, in addition to the patient’s federal identification number, are the main field dependencies of this solution. Once the nurse concludes the defaulted health screening, the patient’s federal identification number is input whereby the digital solution searches for the data linked to this identifier and completes the medical care registration form with all the mandated information for a complete medical record and links it to the queuing system number. This ensures a definitive linkage to the patient’s health record, queuing number after the medical care registration, expediting the patient’s journey immediately to the doctor’s consultation. This solution is installed and used at the nursing workstations by properly trained nursing caregivers [37]. Despite the success stories of using EHR on responsiveness and accuracy, observational studies highlighted a negative impact on patient-physician communication behaviors when physicians are engaged with computers and ignoring their patients [38]. These moments of silence or screen gaze could be due to complicated systems or lack of training. Also, the frustration of physicians with the EHR system may translate into dissatisfaction, resistance to use, and missing some key information [39].

RPA has proven to be the technology of the future, providing sustainable solutions that reduce costs and delivery time, improve quality, and the speed and operational efficiency of business process complexities [7,36]. Additionally, it integrates different systems using software robots to automatically acquire and integrate data from clinical applications, lab information systems, third-party portals, insurance portals, radiology information systems, enterprise resource planning (ERP) and human resources (HR) applications, and scheduling applications. RPA frees employees from the repetitive tasks of scheduling so they can apply their skills to scenarios that require human interaction and touch and automate the eligibility requests to access information for better communication to providers and patients [40]. RPA drastically reduced the amount of time staff spent on repetitive and routine activities from appointment scheduling, hence optimizing appointment scheduling, patient account creation and verification, file handling and processing, inventory management, claims processing, and test management [41].

Another form of digital transformation is in the use of connected ambulances, which are vehicles equipped with advanced communication technology that allows them to transmit vital patient information, such as medical history, vital signs, and real-time video, to health care providers before the patient arrives at the hospital. If care is not ensured in the emergency department, follow-up treatment in the outpatient clinics will be negatively impacted. These ambulances are essentially mobile telemedicine platforms, enabling health care professionals to assess and prepare for incoming patients more effectively [42]. The impact of connected ambulances on service reliability in outpatient clinics can be significant. By providing health care providers with detailed information about incoming patients, including their medical condition and needs, connected ambulances facilitate better preparation and coordination of care upon the patient’s arrival. This can lead to reduced waiting times, more timely interventions, and improved overall patient outcomes. Overall, the use of connected ambulances can help outpatient clinics improve service reliability by streamlining processes, enhancing communication, and optimizing resource allocation to better meet the needs of patients [43].

Tables 3 and 4 depict respondents’ perspectives on the influence of RPA on various organizational aspects, driving its adoption. It also delineates the impact of RPA on quality dimensions (including responsiveness, waiting, and service time), predominantly indicating favorable effects across organizational domains. Regarding connected ambulances, respondents affirmed they would indirectly positively affect responsiveness, waiting times, and service efficiency, as well as other dimensions, except for physicians’ professional competence, credibility, and technical performance.

Table 5 demonstrates a strong alignment between expert opinion and the systematic literature regarding the impact of digital technologies on outpatient health care services. RPA received the highest level of SME consensus (84%), followed by telemedicine (74%) and AI-enabled insurance processing (71%), indicating that experts perceive these technologies as having the greatest potential to improve operational efficiency, reduce waiting times, and streamline administrative processes. Telemedicine also had the strongest evidence base in the literature, followed by EMR and EHR systems and RPA, reflecting substantial research support for their effectiveness. Across technologies, reported benefits consistently included enhanced efficiency, improved patient access, strengthened data integration, better remote monitoring, and increased patient education, although challenges such as digital literacy and usability remained important implementation considerations. Overall, the findings suggest broad agreement between SME perspectives and published evidence regarding the value of digital technologies in improving outpatient health care delivery.

**Table 5.** SME^a^ consensus rates and literature support per technology (19 SMEs and 53 SLR^b^ studies).

Technology	SMEs reporting positive impact (%)	Number of SLR studies supporting	Key outcomes reported
RPA^c^	84	7	↓ Waiting times (20%‐40%), ↑ efficiency
Telemedicine and virtual visits	74	11	↑ Access, ↑ satisfaction, digital literacy required
EMR^d^ and EHR^e^	63	9	↑ Data integration, usability issues noted
Health care wearables	68	6	↑ Remote monitoring, literacy barriers
AI (insurance processing)	71	5	↑ Claim turnaround, ↓ admin time
Educational technology	60	4	↑ Empathy training outcomes
3D anatomy modeling	58	3	↑ Patient education, trust
PDAs^f^ and decision support tools	55	3	↑ Physician decision support

a SME: subject matter expert.

b SLR: systematic literature review.

c RPA: robotic process automation.

d EMR: electronic medical record.

e EHR: electronic health record.

f PDA: personal digital assistant.

### Dimension 2: Reliability (Competence)

Applying reliability principles to health care has the potential to help reduce “defects” in care or care processes, increase the consistency with which appropriate care is delivered, and improve patient outcomes. It is a failure-free operation over time and an individual patient’s experience over time, particularly effectiveness, timeliness, and patient-centeredness. Providing reliably good care over time requires understanding and addressing the reasons that patients are returning to the clinics [44]. In health care, reliability must be built across, not just within, groups and organizations; it has come to be used as a proxy for safety despite reliability and safety not being equivalent [45].

In outpatient clinics, the application of robotics and automation is broadly designated, such as robot areas, to assist patients and disseminate information about various units of the specialized clinic and hospital as a whole. Those robots can handle several visitors without becoming tired, stressed, or overloaded and direct the patient to the correct physician of choice. They are deemed attractive to patients, especially children, enhancing the overall patient experience. Moreover, humanoid nurse robots (HNRs) are being used to care for older adult patients, offering 24/7 and 7/7 service with minimal cost, inclusive of lifting and therapeutic assistance. Telemedicine robots are helpful in telemedicine applications where a virtual doctor takes all the physiological parameters and diagnoses a disease using audiovisual aids. Those systems are extremely useful in the case of pandemics whereby the patients are located in remote areas and cannot reach physicians for clinical diagnosis and treatment; those robots are used as a low-cost remote interface [46]. Vendors have developed sophisticated remote-controlled cars with advanced cameras, lenses, and connected microphones, which enable the physician to interact with the patient’s bedside despite being miles away [47]. Nowadays, different versions of telemedicine such as online health platforms [48] and remote presence virtual and independent telemedicine assistants are widely used by hospitals [49].

Furthermore, an on-demand health care solution refers to a service or platform that provides immediate access to medical care and resources whenever and wherever needed. This could include telemedicine platforms, virtual consultations, remote monitoring devices, or mHealth apps. On-demand health care solutions leverage technology to connect patients with health care providers in real-time, offering convenience, accessibility, and timely assistance [50].

Tables 3 and 4 illustrate the viewpoints of participants regarding how telemedicine and virtual visits influence different organizational aspects, prompting their acceptance of this technology. It outlines the impact of these advancements on 9 quality measures, such as service reliability and assurance, indicating predominantly positive effects across organizational realms. Nonetheless, respondents noted that while telemedicine enhances most quality measures, it falls short in improving data collection capabilities and demands patients to possess technological proficiency for maximal benefits. Moreover, some studies suggest that reducing the visit length using technology is associated with medical errors such as inappropriate antibiotic prescriptions [51]. Conversely, on-demand health care solutions are seen to positively affect all quality measures.

Integrating technical support services with the implementation of EMRs in health care outpatient centers and clinics is essential for ensuring smooth operational performance, maintaining system reliability, and promptly addressing any technical challenges that may arise during system use [52]. Technical support teams play a fundamental role throughout the lifecycle of EMR deployment, beginning with the initial implementation phase and continuing through ongoing system optimization and maintenance.

During the early stages of EMR implementation, technical support teams provide critical assistance with system installation, configuration, and data migration. Their specialized expertise ensures that health care organizations can transition from traditional record systems to digital platforms with minimal disruption to clinical workflows and patient care processes. By carefully managing these technical transitions, support teams help reduce operational interruptions and facilitate a smoother adoption of EMR systems across health care facilities [53].

In addition to implementation support, technical teams are responsible for conducting training sessions and developing educational materials that help health care professionals effectively use EMR systems. These training initiatives typically include guidance on accurate data entry, system navigation, and the use of advanced system functionalities. Well-trained health care personnel are better able to use EMR capabilities efficiently, which contributes to improved accuracy, enhanced documentation quality, and more efficient patient care delivery [54].

Technical support teams also play a key role in the day-to-day operation of EMR systems by providing timely troubleshooting services and resolving technical issues that may occur during routine clinical use. Their responsibilities often include performing regular system maintenance tasks such as applying software updates, installing security patches, and optimizing system performance to ensure reliability and data protection. Furthermore, these teams collaborate closely with health care providers to customize EMR systems according to specific clinical workflows and operational requirements. They also facilitate the integration of EMR systems with other health care information technologies, including laboratory information systems and picture archiving and communication systems (PACS). Such integration enhances interoperability between different health care platforms, enabling more efficient data exchange and improving the overall effectiveness of health care delivery [55,56].

Another critical function of technical support teams involves maintaining the security and regulatory compliance of EMR systems. Health care organizations must adhere to strict data protection regulations, including standards such as the Health Insurance Portability and Accountability Act (HIPAA). Technical teams therefore implement comprehensive security mechanisms, including access control protocols, data encryption, and periodic security audits to safeguard sensitive patient information from unauthorized access or potential breaches. Ensuring regulatory compliance not only protects patient privacy but also helps health care organizations avoid legal and financial risks associated with data security violations [57].

To ensure continuous system availability, many technical support providers also offer 24-hour helpdesk services and emergency assistance. This round-the-clock technical support is particularly important in health care environments where uninterrupted access to patient data is essential for timely clinical decision-making. Rapid response to system failures or technical issues helps minimize downtime and ensures that health care professionals can access critical patient information whenever needed, thereby supporting effective clinical care and patient safety [57].

Beyond operational support, technical teams continuously monitor the performance and usage patterns of EMR systems to identify opportunities for improvement. They gather feedback from health care staff and collaborate with EMR vendors to implement system updates, enhancements, and new functionalities that align with evolving health care standards and best practices. Through this ongoing monitoring and system refinement process, health care organizations can maintain efficient, reliable, and secure EMR infrastructures. Ultimately, the integration of dedicated technical support services with EMR implementation and maintenance significantly enhances the effectiveness of EHR systems and contributes to improved health care quality and patient outcomes [58].

The adoption of EMRs has significantly transformed health care delivery by improving operational efficiency, documentation accuracy, and accessibility of patient information. As health care systems increasingly transition from paper-based records to digital platforms, EMR systems offer multiple advantages that enhance both clinical and administrative functions within health care organizations.

One of the primary benefits of EMR implementation is the streamlining of administrative tasks. Processes such as patient record management, documentation, and prescription handling become more efficient through digital systems, allowing health care providers to reduce time spent on manual paperwork. As a result, clinicians can allocate more time and attention to direct patient care, ultimately improving the quality of health care services provided [59].

EMRs also provide a comprehensive and accurate overview of a patient’s medical history, enabling health care professionals to access complete and organized clinical information in a timely manner. This improved accessibility supports more informed clinical decision-making and reduces the likelihood of medical errors that may arise from illegible handwriting, incomplete documentation, or fragmented paper records. By ensuring that patient data are clearly documented and readily available, EMRs contribute to safer and more reliable health care delivery [60].

In addition, EMR systems facilitate remote access to patient records, which significantly enhances communication and collaboration among health care providers. Physicians, specialists, and other health care professionals can access patient information from different locations, allowing for faster consultations, coordinated care, and improved clinical outcomes [61]. Patient engagement is also strengthened through EMR-integrated patient portals, which enable individuals to view their medical information, communicate electronically with health care providers, and manage appointments through online platforms. These features empower patients to become more actively involved in managing their own health and health care decisions [62].

Furthermore, the digital nature of EMR systems allows health care organizations to aggregate and analyze large volumes of clinical data. This capability supports advancements in population health management, enables more robust clinical research, and facilitates quality improvement initiatives across health care systems. By leveraging EMR-generated data, health care providers and policymakers can identify health trends, evaluate treatment outcomes, and implement strategies that enhance the overall effectiveness and efficiency of health care delivery.

While initial implementation costs can be substantial, long-term cost savings are realized through increased efficiency, reduced paperwork, and better management of chronic conditions [63]. Furthermore, the interoperability of EMRs improves continuity of care by enabling seamless sharing of patient information between different health care providers and systems [60]. Enhanced compliance with regulations such as HIPAA and robust security measures ensures the protection of patient data, further bolstering the benefits of digital transformation in health care using EMRs [64].

Tables 3 and 4 illustrate the perspectives of respondents regarding the influence of EMR on different organizational aspects, motivating their interest in adopting this technology. It also outlines the effects of EMR systems on quality dimensions, such as technical support services, which show improvement with the digitization of medical records and related clinical operations. This primarily indicates a positive impact across various organizational sectors.

### Dimension 3: Environment of Service Delivery (Cleanliness)

Cleaning and disinfection of surfaces in clinics are strategies to minimize the occurrence of health care–associated infections. Given that clinics with it increase patient volumes are environments that act as a pool for bacteria and viruses that may spread in the clinic causing infectious disease. For stringent infection control, an intensified cleaning and disinfection process is applied [65]. Moreover, strict policies and procedures to eliminate cross-contamination in outpatient clinics are essential [66], such as disposable glove changes, caregiver’s hand washing, clinic surfaces being disinfected often, tools and equipment cleaning and sterilization, and single-use supplies disposal.

Cleaning robots are used in outpatient clinics; they use intelligent navigating dry vacuum pumps for dry or wet mopping activities, being an integral part of disinfecting the physical space from germs and pesticides. Moreover, the UV radiation-based robot is used to disinfect the clinic from microbes and clean washrooms using sensing systems. Spraying and disinfection robots widely spread antiseptic combinations over large indoor and outdoor areas [46].

Tables 3 and 4 depict the respondents’ views on how robotics affects various organizational aspects, driving their inclination toward adopting this technology. It also delineates the impact of robotics on quality dimensions, including the environment of delivering service, primarily indicating a favorable influence across organizational sectors. Robotics enhances surgical precision (outcome of surgery), resulting in expedited recovery and rehabilitation, thereby improving surgical outcomes.

### Dimension 4: Communication

Interpersonal communication plays a key role in the patient’s treatment plan; not only does it impact patient satisfaction levels with the offered clinical service but also it places them in the role of a partner in the journey building an environment of empathy, friendliness, kindness in treatment, safety, and trust. Enhanced communication guarantees that patients have access to their health information and ultimately enhances the quality of patient care within an outpatient clinic setting [67]. Moreover, communication errors can be a profound contributing factor in outpatient clinic sentinel events resulting in poor health outcomes alongside elevated distress levels in patients and their family members. Effective communication skills are deemed a core clinical skill expected from every clinical caregiver when effectively exchanging information between a patient and them and include communication with the family and carer as well. Among the key elements of effective communication in clinics is the ability to foster relationships, the 2-way exchange of information, convey empathy, engage patients in decision-making and care planning, and manage complexity and uncertainty [68].

Patient preferences are leaning toward using technology when communicating about their symptoms with their health providers in clinics especially after discharge from outpatient clinics. Email communication, phone, text messaging, mHealth portal apps, social media, online discussion forums, and videoconferencing [69]. Furthermore, patient education is a crucial element within health care. It is known as forecasters for increased patient engagement levels in shared decision-making, improved medication and treatment adherence, higher levels of satisfaction, and even better overall treatment outcomes. Most patients are simply not capable of processing large amounts of new medical information in a short period of time. Apps for smartphones and tablets have the potential to actively educate patients by providing them with timely information using push notifications [70]. Finally, Owens et al [71] conducted a study to evaluate the use of ambient voice recognition, coupled with natural language processing and AI in patient-physician relationships. The feedback of 288 patients suggests a strong positive correlation between the use of this technology and patient-physician communication, which improved patients’ satisfaction.

The impact of on-demand health care solutions on the communication quality dimension is significant. First, these solutions facilitate seamless communication between patients and health care providers, overcoming barriers such as geographical distance or time constraints. Patients can easily connect with health care professionals via video calls, chat messaging, or phone calls, enabling timely access to medical advice and assistance [72]. Moreover, on-demand health care solutions often incorporate features that enhance the quality of communication. These may include secure messaging platforms, multimedia capabilities for sharing images or documents, and real-time data exchange functionalities. By providing clear channels for communication, these solutions enable patients to effectively convey their symptoms, concerns, and medical history to health care providers, leading to more accurate diagnoses and personalized treatment plans [73]. Additionally, on-demand health care solutions can improve communication among health care teams. They enable seamless collaboration and information sharing between primary care providers, specialists, and other health care professionals involved in a patient’s care [74]. This interdisciplinary communication fosters coordinated and integrated health care delivery, ultimately improving patient outcomes [75].

Tables 3 and 4 present the viewpoints of the respondents regarding the impact of mHealth portal apps and videoconferencing on various organizational aspects, which motivate their adoption of these technologies. It also delineates the effects of these apps on quality dimensions, notably communication, predominantly indicating a significantly positive influence across organizational sectors. Particularly for mHealth portal apps, an equal number of respondents expressed a perception that, unlike their counterparts, they observe no impact on physicians’ professional competence, credibility, technical performance, as well as patient-centeredness and empathy (staff demeanor). However, for the sake of this research, the favorable responses were taken into consideration.

### Dimension 5: Empathy (Staff Demeanor)

Terry and Cain [76] stressed the criticality of empathy throughout the patient care journey and optimal patient-caregiver relationship. It increases both the patient’s satisfaction and compliance and elevates the caregiver’s ability to cater to the clinical needs of their patients. Furthermore, it has a strong correlation and positive impact on the health outcomes of patients and decreases the risk of any potential malpractice liability. Empathy in its core element does not change yet evolves to align with the digital advancements in the channels of provisioning and communicating clinical services.

With the widespread use of telemedicine in outpatient clinics, the construct of digital empathy has become an increased necessity and must be integrated into the academic and training curricula of health professionals [76]. An increasing trend in the use of digital health tools to engage patients and enhance outcomes is seen; with its effectiveness being dependent on the consistency of the patients’ use and engagement, with empathy being the foundation of such tools. This has further increased the importance of not only the caregiver’s increase in their telemedicine levels but also the importance of architects being more considerate with their home designs, especially with working from home being the future of work, especially within the lifespan; the same homes may have to accommodate chronically ill patients. Hence, making the design of sustainable and healthy homes a new trend, that is, designing for well-being in residential health care. It is more than having a healthy physical body and includes social, spiritual, emotional, and intellectual well-being. Incorporating nature into the environment through biophilic design creates a sense of serenity, therapeutic, and well-being effect in the homes of patients [77].

Tables 3 and 4 provide insights into how educational technology influences various aspects within outpatient clinics, shedding light on why these clinics are increasingly inclined to adopt such technology. The findings suggest that educational technology has a predominantly positive effect across different areas within outpatient clinics, indicating its potential to enhance patient care, staff interactions, and overall clinic operations. This underscores the importance and potential benefits of integrating educational technology into outpatient clinic settings, not only for improving efficiency and effectiveness but also for fostering positive patient experiences and enhancing the quality of care provided through more empathy levels by the care providers [78]. Tables 3 and 4 depict respondents’ perspectives on the influence of RPA on various organizational aspects, driving its adoption. Respondents observed that while RPA improves most quality dimensions, it does not significantly enhance patient-centeredness or empathy (staff demeanor). It also delineates the impact of RPA on quality dimensions (including responsiveness, waiting, and service time), predominantly indicating favorable effects across organizational domains. Such findings are in line with the study by Chen et al [79], who suggested that efficiency improvement that results in shorter visit duration can negatively impact the provision of counseling and the patient-physician relationship and communication.

### Dimension 6: Nurse Care

According to the study by Oermann and Templin [80], patients perceive high nursing quality when they are cared for by nurses who are knowledgeable and resourceful. Having direct access to the nurse and can communicate and spend enough time not being rushed during the clinic visit. Moreover, they consider the fact that a nurse educates them and teaches them more about their illness (especially among those who are less educated, have lower income levels, and have been living with chronic illness) and the medications that they require; reiterating the treatment plan and expectation setting alongside coaching them on how to remain healthy was a quality differentiating factor. Additionally, having the ability to reach out at any given time for clarifications, concerns, or guidance is becoming more critical than ever.

Patients are expecting more detailed explanations about their health situation to increase their understanding and minimize their anxiety levels and complaints; making the use of 3D digital anatomy modeling an aid to teaching and its use in patient education and operation planning. Clinicians will be expected to ensure that the information is retained and risks and benefits weighed up in the 3D computer animation. Patients’ understanding improved, their trust levels increased in the professional treatment, and their anxiety reduced toward their medical conditions and treatment plans [81].

Tables 3 and 4 illustrate the opinions of participants regarding the influence of 3D digital anatomy modeling on different organizational facets, motivating their interest in embracing this technology. It also outlines how this technology affects 9 quality aspects, including nursing care, mostly suggesting a positive effect across organizational domains and that it permits a more effective 2-way dialogue with the patient. Nonetheless, respondents noted that for 4 dimensions, they observed no impact of this technology on (1) improving productivity, (2) physicians’ professional competence, credibility, and technical performance, (3) responsiveness and waiting and service time, and (4) access to care and health care inequality.

### Dimension 7: Access to Care and Health Care Equality

Access to medical care is a comparatively multifaceted phenomenon categorized by potential access and realized access. The latter is what matters in outpatient clinics, as it resembles the use of physician time for all specializations, which makes the design of robust policies to optimize this quality determinant. If patients can’t reach care, their satisfaction levels will drop negatively, impacting their treatment journey [82].

Digital transformation improves access to health care services and supports health insurance processing large numbers of claims and payments, which upscales the service of outpatient clinics [83]. Davenport and Kalakota [36] pointed out that machine learning, neural networks, deep learning, and precision medicine are where AI predicts what treatment protocols are likely to succeed for a patient based on various patient attributes and his or her treatment context. In addition, Bruce and Hardy [84] added that the use of natural language processing (NLP) is where AI involves the creation, understanding, and classification of clinical documentation and published research, viewing disease history and analyzing unstructured clinical notes on patients, preparing reports, and transcribing patient interactions. An example is IBM’s Watson and Google DeepMind Health. AI combined with consumer wearables and other medical devices is applied to oversee early stages of heart diseases, allowing caregivers to monitor and detect potentially life-threatening episodes at an early stage.

Tables 3 and 4 present the perspectives of respondents concerning the impact of AI in insurance processing on various organizational aspects, stimulating their inclination toward adopting this technology. It also delineates the influence of this technology on 9 quality dimensions, with particular emphasis on access to care and health care inequality, generally indicating a positive impact across organizational realms. It is noteworthy that some respondents highlighted its tendency to diminish the soft skills of insurance professionals despite its benefits. Regarding health care wearables, participants affirmed that they observed a beneficial influence across various quality aspects except for patient-centeredness and empathy (staff behavior) and responsiveness, waiting, and service time, as they believe these dimensions remain unaffected by this technology. One main challenge to information-access equality is patients’ digital literacy and access. Negative attitudes toward technology, disinterest in learning new skills, physical and mental disability, and financial hardships are among the reasons for technology avoidance [85]. Such challenges may undermine the benefits gained from technological adoption, such as telemedicine, and make it a curse rather than a blessing.

### Dimension 8: Physician’s Professional Competence, Credibility, and Technical Performance

This dimension is concerned with the physician consultation stage of the patient journey in terms of the way the doctor diagnoses and explains the treatment plan to the patient, reflecting their competency level as a key determinant of quality for their clientele. Moreover, leadership training, systems theory and analysis, cross-disciplinary training and multidisciplinary teams, understanding and respecting the skills of other practitioners, population health management, palliative care and end-of-life and resource management and medical economics, health policy and regulation, less ‘‘captain of the ship’’ and more ‘‘member/leader of the team,’’ empathy and customer service, time management, conflict management, providing performance feedback, understanding cultural and economic diversity, and emotional intelligence were identified as the core physician skills required for the next generation of health care delivery [86].

Medical information technology throughout the continuum of the physician’s lifelong journey has become a widespread phenomenon providing physicians access to teleconference podcasts, consulting resources in PDAs for enhanced patient management decisions at the point of care. Medical simulators are another form of technology that is also increasingly being used for targeted training and assessment that would elevate the technical skill set levels of care providers [87]. A reliable disease history analysis is integral to assessing a physician’s professional competence, credibility, and technical performance. Physicians who possess a thorough understanding of their patients’ medical histories demonstrate competence in their ability to gather and analyze pertinent information critical to diagnosis and treatment decisions. This competence enhances their credibility among patients and colleagues alike, fostering trust and confidence in their clinical judgment [88]. Furthermore, a meticulous disease history analysis enables physicians to demonstrate technical proficiency in identifying relevant patterns, risk factors, and potential complications associated with a patient’s health status. By effectively using this information, physicians can deliver more precise and tailored care, thereby enhancing their overall performance and ensuring optimal patient outcomes [89]. Thus, a reliable disease history analysis is not only essential for providing high-quality health care but also for upholding the standards of professional competence, credibility, and technical proficiency expected of physicians [90].

Tables 3 and 4 showcase respondent viewpoints regarding the effects of PDAs on insurance processing across organizational facets, encouraging proclivity toward its adoption. It outlines the impact of PDAs on 9 quality dimensions, notably focusing on physician’s professional competence, credibility, and technical performance, generally indicating a positive influence across organizational domains, except for patient-centeredness and empathy, where respondents perceived no effect from this technology. Notably, some respondents pointed out the low quality of data collected by PDAs and that using PDAs dilutes accountability and responsibility. In examining disease history, participants confirmed a positive impact across all dimensions except for the environment of delivering service (cleanliness). Conversely, in evaluating teleconference podcast technology, all dimensions showed a positive effect except for responsiveness, waiting times, and service efficiency.

In summary, each technology comes with benefits and some negative impact on some of the quality dimensions. Training and good change-management practices can help overcome these negative aspects and better integrate the technology into the patient care system [91]. Table 4 provides a summary of the positive and the negative impacts of technologies adopted in health care based on the literature review and SMEs’ feedback.

The study is limited by its qualitative nature and reliance on SME perspectives, which may not generalize across all outpatient contexts. Additionally, limited patient-reported outcomes restrict conclusions on direct patient experience impact. Quantitative evaluations using patient satisfaction surveys, randomized trials of specific digital tools, and cost-effectiveness analyses are needed. Further exploration into digital literacy interventions and human-centered technology design is recommended to optimize patient-centered outcomes.

This systematic review contributes to the literature by providing an integrated synthesis of how emerging digital health technologies, including telemedicine, wearable monitoring, and clinical automation, collectively influence PCQ in outpatient settings. The review is innovative in its holistic approach, moving beyond technology-specific evaluations that dominate existing reviews and instead examining the combined impact of multiple digital health modalities within a unified analytical framework. By organizing and interpreting evidence across fragmented research streams, the study advances theoretical understanding of digital transformation in health care and clarifies the pathways through which digital tools enhance accessibility, efficiency, and patient-centered care delivery. In practical terms, these findings offer actionable insights for health care leaders, policymakers, and technology developers seeking to implement digital solutions that improve service quality and patient outcomes in real-world outpatient care environments.

Taken together, the findings demonstrate that emerging digital technologies influence outpatient service quality through multiple operational and clinical pathways. Automation technologies primarily improve efficiency-related dimensions such as responsiveness and workflow coordination, while digital communication tools strengthen patient-provider interaction and access to care. In contrast, technologies that rely heavily on patient engagement, such as telemedicine platforms and wearable monitoring devices, reveal the importance of digital literacy and user-centered system design. These findings align with recent literature suggesting that digital transformation in health care improves operational performance but requires complementary organizational strategies to fully enhance patient-centered care. The results therefore reinforce the importance of integrating technological innovation with training, workflow redesign, and patient engagement strategies to achieve meaningful improvements in health care quality.

Despite its contributions, this study has several limitations that should be considered when interpreting the findings. First, the systematic review was limited to studies published in English between 2017 and 2022, which may exclude relevant research published in other languages or earlier foundational studies. Second, the review synthesized evidence across different health care systems and geographical contexts, which may limit the generalizability of specific findings to all outpatient settings. Third, although expert interviews provided valuable contextual insights, the sample size of 19 participants may not capture the full diversity of perspectives across the health care industry. Additionally, some technologies included in the analysis are evolving rapidly, meaning that their impacts on PCQ may continue to change as implementation practices mature. Finally, the study primarily relied on qualitative synthesis rather than meta-analysis due to heterogeneity across study designs and outcome measures, which limits the ability to quantify the magnitude of technology effects on PCQ.

The findings of this review have several implications for both research and health care practice. From a theoretical perspective, the study demonstrates the value of applying service quality frameworks such as SERVQUAL to evaluate the impact of digital transformation on health care delivery. By integrating evidence across multiple technology domains rather than examining individual innovations in isolation, this review provides a more comprehensive understanding of how digital health ecosystems influence PCQ. From a practical standpoint, the results highlight that health care organizations should approach technology adoption as part of a broader transformation strategy that includes workforce training, workflow redesign, and patient engagement initiatives. Future research should further investigate how different technologies interact within health care systems and explore longitudinal evidence on their long-term effects on patient outcomes and health care equity. Ultimately, the effective integration of emerging digital technologies has the potential to strengthen outpatient care delivery and support more responsive, accessible, and patient-centered health care systems.

### Conclusion

Using the augmented SERVQUAL model, the analysis presented in this research offers a structured evaluation of various emerging technologies’ impacts on organizational aspects within health care and insurance processing domains. Each technology is assessed across 9 quality dimensions, enabling a comprehensive understanding of its effects on service quality, efficiency, and patient care. The findings reveal a predominantly positive influence of technologies such as RPA, telemedicine, robotics, EMR, mHealth portal apps, educational technology, 3D digital anatomy modeling, AI in insurance processing, health care wearables, PDAs, and teleconference podcast technology on organizational performance. These technologies demonstrate strengths across dimensions such as service reliability, communication, and technical support services.

Patients differ significantly in their attitudes and preferences; some may be submissive, readily accepting prescribed treatments, while others might be technology enthusiasts who prefer to engage with digital health tools. Additionally, there are those who might challenge medical advice and seek more information or alternative options. Recognizing and addressing these variations is crucial for delivering personalized and effective care, especially if technology is used [34]. Although automation is an important enabler for productivity boost, it treats every patient the same way, ignoring the fact that patients have diverse expectations and needs.

However, nuanced insights emerge from the analysis. While most technologies exhibit positive effects, challenges such as data quality issues, technological proficiency requirements for users, and limited impact on certain dimensions are identified. For example, while telemedicine enhances service reliability and accessibility, it may require patients to possess adequate technological skills for optimal use. For example, the use of EHR to reduce patient visits, coupled with poor usability of the system, which results in physicians’ distraction, may negatively impact patient-centeredness. Proper integration of EHRs in clinical practice may boost the patient-physician communication dynamics, while poor usability and long keyboarding may jeopardize this communication. Moreover, although technology adoption provided significant reductions in visit and waiting time, shorter visit time may be associated with medical errors and lack of patient-centeredness. As a result, optimal visit time is necessary. Table 6 summarizes the components of the literature search strategy, including the databases searched, the timeframe covered, the language restriction, and the study types included in the review.

Table 7 summarizes the potential positive and negative impacts of the technologies identified in the literature, organized according to health care service quality dimensions and supported by evidence from the included studies.

**Table 6.** Components of the literature search.

Component	Details
Databases searched	ABI/INFORM Global, ScienceDirect, Medline (via PubMed), and Google Scholar
Timeframe	January 2017-December 2022 (SERVQUAL^a^ foundational literature included up to 1992)
Language	English
Study types	Peer-reviewed empirical studies and systematic reviews

a SERVQUAL: Service Quality Model.

**Table 7.** Summary of potential impact of technologies.

Dimension and technology	Negative impact	Positive impact
Responsiveness		
EHR^a^	Missing some key information due to EHR literacy and usability [39]	Appointment scheduling [35] Data collection accuracy [37]
RPA^b^	—^c^	Waiting time reductions [36] Better communication to providers and patients [40] Improve efficiency and use [41]
Connected ambulances	—	Improve incoming patients’ treatment effectiveness [42] Enhance communication and use [43]
Reliability		
HNR^d^	—	Improve diagnoses and care [46]
Telemedicine	Visit length reduction may increase medical errors such as inappropriate antibiotic prescription [51] Reduction of data collection (SMEs^e^)	Improve health accessibility and improve use [47-49]
EMR^f^	—	Maximizing system uptime and addressing issues that may arise [52,54] Enhancing data security [57,64] Enabling seamless sharing of patient information between different providers and systems [60]
On-demand health care	—	Improve convenience, accessibility, and timely assistance [50]
Robotics	—	Enhances surgical precision resulting in expedited recovery and rehabilitation
Cleanliness		
Cleaning robots	—	Intensified cleaning and disinfection improvement [46,65]
Communication		
EHR	Lack of patient-physician communication behaviors due to data entry and keyboarding distraction [38]	Enhanced health information accessibility and overall PCQ^g^ [67] Patient engagement in decision-making [68]
Videoconferencing	—	Increased patient engagement and enhanced treatment outcomes [69,72,73]
Apps	Shorter visit duration can negatively impact provision of counseling and the patient-physician relationship and communication [79]	Improve patients’ education and timely information notifications [70]
Empathy		
Telemedicine	—	Enhance patients’ engagement
Voice recognition	—	Improve patient-physician communication [71]
RPA	No significant impact on empathy (SMEs)	—
Nurse care		
3D computer animation	—	Increase trust levels reduce anxiety [81]
Access to care		
AI in health insurance, mobile apps, and wearables	Digital illiteracy results in a negative attitude toward technology [85] Diminishing the soft skills of insurance professionals (SMEs)	Improve serviceability and satisfaction [36,83]
Professional competence		
Medical simulators	—	Elevate the technical skill set levels of care providers [87]
PDA^h^ and EMR	Low quality of data collected by PDAs dilutes accountability and responsibility (SMEs)	Fostering trust and confidence in care providers’ clinical judgment [88-90]

a EHR: electronic health record.

b RPA: robotic process automation.

c Not available.

d HNR: humanoid nurse robot.

e SME: subject matter expert.

f EMR: electronic medical record.

g PCQ: patient care quality.

h PDA: personal digital assistant.

The SERVQUAL model facilitates a systematic examination of these technologies’ impacts, enabling stakeholders to identify areas of strength and opportunities for improvement. By addressing challenges and leveraging strengths, organizations can enhance their adoption and implementation of emerging technologies, thereby optimizing service delivery, patient care, and operational efficiency within health care and insurance processing sectors.

Despite extensive efforts to identify and evaluate the determinants influencing digital transformation in health care, existing literature provides limited evidence regarding the measurable value, temporal evolution, and relative prioritization of these determinants, particularly from the patient perspective. Current research has largely focused on conceptual assessments and implementation considerations, while the empirical relationship between digital initiatives, return on investment, service quality, and human-centered care remains insufficiently explored. Therefore, future research should adopt quantitative and mixed methods approaches to validate these determinants, assess their impact over time, and establish evidence-based frameworks that balance technological advancement with patient expectations, operational efficiency, and quality of care.

## Supplementary material

10.2196/81847 Checklist 1 PRISMA 2020 checklist.

10.2196/81847 Checklist 2 COREQ checklist.

## Abbreviations

COPQ: cost of poor quality
COREQ: Consolidated Criteria for Reporting Qualitative Research
EHR: electronic health record
EMR: electronic medical record
ERP: enterprise resource planning
HIPAA: Health Insurance Portability and Accountability Act
HNR: humanoid nurse robot
HR: human resources
IaaS: Infrastructure as a Service
IoT: Internet of Things
IRB: Institutional Review Committee
mHealth: mobile health
NLP: natural language processing
PaaS: Platform as a Service
PACS: picture archiving and communication systems
PCQ: patient care quality
PDA: personal digital assistant
PRISMA: Preferred Reporting Items for Systematic Reviews and Meta-Analyses
PRISMA-S: Preferred Reporting Items for Systematic Reviews and Meta-Analyses Literature Search Extension
RPA: robotic process automation
SaaS: Software as a Service
SERVQUAL: Service Quality Model
SLR: systematic literature review
SME: subject matter expert
