# Supplementary material for: Impact of Emerging Technologies on Outpatient Care Quality: Mixed Methods Study
Source: JMIR Rehabil Assist Technol. 2026 Aug 19;13:e81847. doi: 10.2196/81847 (PMC13494525; doi:10.2196/81847)
Supplement: Supplementary file 2 [file rehab-v13-e81847-s001.docx]

**PRISMA 2020 Checklist – Completed for the Manuscript**

| **Section and Topic** | **Item #** | **PRISMA Item Description** | **Location in Manuscript** | **Other** |
| --- | --- | --- | --- | --- |
| TITLE | 1 | Identify the report as a systematic review in the title. | Title page identifies the work as a systematic review examining the impact of emerging healthcare technologies on outpatient care quality. |  |
| ABSTRACT | 2 | Provide a structured abstract following the PRISMA for Abstracts checklist. | Abstract section | Structured abstract follows PRISMA guidance. |
| INTRODUCTION - Rationale | 3 | Describe the rationale for the review in the context of existing knowledge. | Introduction section discussing the growing adoption of digital technologies in healthcare and their influence on patient care quality. |  |
| INTRODUCTION - Objectives | 4 | Provide an explicit statement of the objectives or research questions. | Final paragraph of the Introduction | Research questions specify how emerging technologies influence outpatient care quality and organizational objectives. |
| METHODS - Eligibility Criteria | 5 | Specify inclusion and exclusion criteria and grouping of studies. | Methods section describing study eligibility criteria and systematic review inclusion framework. | Grouping of studies based on technology categories and healthcare service quality dimensions. |
| METHODS - Information Sources | 6 | Specify databases, registers, websites, and sources searched, including the last search date. | Methods section and Multimedia Appendix 1 | Databases searched include major healthcare and management databases used for systematic review retrieval. |
| METHODS - Search Strategy | 7 | Present full search strategies for databases and sources. | Multimedia Appendix 1 | Full search strings provided in supplementary material. |
| METHODS - Selection Process | 8 | Describe screening process and number of reviewers involved. | Methods section describing PRISMA screening process. | Study screening followed PRISMA guidelines for record identification and eligibility. |
| METHODS - Data Collection Process | 9 | Describe how data were extracted and by whom. | Methods section describing literature extraction and SME interview integration. |  |
| METHODS - Data Items (Outcomes) | 10a | List and define outcomes for which data were sought. | Methods and Results sections | Outcomes focused on patient care quality dimensions such as reliability, responsiveness, and communication. |
| METHODS - Data Items (Other Variables) | 10b | List other variables collected (e.g., study characteristics). | Methods section | Includes study context, technology type, and organizational objectives. |
| METHODS - Risk of Bias Assessment | 11 | Specify methods used to assess risk of bias. | Methods section | Formal risk-of-bias scoring tools were not applied due to heterogeneity of study designs. |
| METHODS - Effect Measures | 12 | Specify effect measures used in the synthesis. | Not applicable to quantitative synthesis | The review employs qualitative narrative synthesis rather than statistical effect measures. |
| METHODS - Synthesis Methods (Eligibility for synthesis) | 13a | Describe processes used to determine eligibility for synthesis. | Methods section | Studies meeting eligibility criteria were included in the narrative synthesis. |
| METHODS - Preparing Data for Synthesis | 13b | Describe methods used to prepare data. | Methods section | Data were extracted and categorized according to SERVQUAL dimensions and technology types. |
| METHODS - Tabulation and Graphical Methods | 13c | Describe tabular or graphical presentation methods. | Results section tables | Results presented through summary tables and thematic synthesis. |
| METHODS - Statistical Synthesis Methods | 13d | Describe statistical synthesis methods or explain why meta-analysis was not performed. | Methods section | Meta-analysis was not conducted due to heterogeneity in study design, outcomes, and reporting metrics. Narrative synthesis was applied. |
| METHODS - Exploration of Heterogeneity | 13e | Describe methods used to explore heterogeneity. | Results discussion | Variation across studies explored through thematic comparison of technologies and service quality dimensions. |
| METHODS - Sensitivity Analyses | 13f | Describe sensitivity analyses conducted. | Not conducted | Sensitivity analyses were not applicable due to the qualitative synthesis design. |
| METHODS - Reporting Bias Assessment | 14 | Describe methods used to assess reporting bias. | Methods section | Reporting bias formally assessed through narrative evaluation of study completeness. |
| METHODS - Certainty Assessment | 15 | Describe methods used to assess certainty in evidence. | Methods section | Formal GRADE assessment not applied; interpretive reliability strengthened through triangulation with SME interviews. |
| RESULTS - Study Selection | 16a | Report results of search and selection process using a flow diagram. | PRISMA Flow Diagram in Results section |  |
| RESULTS - Excluded Studies | 16b | Cite studies excluded after full-text review and reasons for exclusion. | Supplementary materials |  |
| RESULTS - Study Characteristics | 17 | Present characteristics of included studies. | Results section and tables summarizing included studies. |  |
| RESULTS - Risk of Bias in Studies | 18 | Present risk-of-bias assessments for each study. | Not formally scored | Due to methodological heterogeneity, risk-of-bias was discussed qualitatively. |
| RESULTS - Results of Individual Studies | 19 | Present summary results of individual studies. | Results section narrative synthesis and summary tables. |  |
| RESULTS - Results of Syntheses | 20a | Summarize characteristics of studies contributing to synthesis. | Results section synthesis discussion. |  |
| RESULTS - Statistical Syntheses | 20b | Present results of statistical synthesis. | Not applicable | No meta-analysis performed. |
| RESULTS - Heterogeneity Investigations | 20c | Present results of heterogeneity analyses. | Results discussion | Variability interpreted through thematic comparison. |
| RESULTS - Sensitivity Analyses | 20d | Present results of sensitivity analyses. | Not applicable | Sensitivity analysis not conducted. |
| RESULTS - Reporting Bias | 21 | Present assessments of reporting bias. | Discussion section |  |
| RESULTS - Certainty of Evidence | 22 | Present certainty of evidence assessment. | Discussion section | Evidence strength discussed narratively. |
| DISCUSSION - Interpretation | 23a | Interpret results in context of existing evidence. | Discussion section |  |
| DISCUSSION - Limitations of Evidence | 23b | Discuss limitations of evidence included. | Limitations section |  |
| DISCUSSION - Limitations of Review Process | 23c | Discuss limitations of review methods. | Limitations section |  |
| DISCUSSION - Implications | 23d | Discuss implications for research, practice, and policy. | Conclusion section |  |
| OTHER - Registration | 24a | Provide registration details or state not registered. | Methods section | Review was not registered in PROSPERO. |
| OTHER - Protocol | 24b | Indicate where protocol can be accessed. | Methods section | No formal protocol publicly registered. |
| OTHER - Amendments | 24c | Describe amendments to protocol. | Not applicable | No protocol amendments. |
| OTHER - Support | 25 | Describe funding sources and roles. | Acknowledgements section |  |
| OTHER - Competing Interests | 26 | Declare competing interests. | Declarations section |  |
| OTHER - Availability of Data and Materials | 27 | Report availability of data, code, and materials. | Supplementary materials | Data available within manuscript and appendices. |
